# Differential PfEMP1 Expression Is Associated with Cerebral Malaria Pathology

**DOI:** 10.1371/journal.ppat.1004537

**Published:** 2014-12-04

**Authors:** Dumizulu L. Tembo, Benjamin Nyoni, Rekah V. Murikoli, Mavuto Mukaka, Danny A. Milner, Matthew Berriman, Stephen J. Rogerson, Terrie E. Taylor, Malcolm E. Molyneux, Wilson L. Mandala, Alister G. Craig, Jacqui Montgomery

**Affiliations:** 1 Malawi-Liverpool-Wellcome Trust Clinical Research Programme, College of Medicine, Blantyre, Malawi; 2 Department of Parasitology, Liverpool School of Tropical Medicine, Liverpool, United Kingdom; 3 Blantyre Malaria Project, College of Medicine, Blantyre, Malawi; 4 Department of Pathology, Brigham and Women's Hospital, Boston, Massachusetts, United States of America; 5 Department of Immunology and Infectious Disease, Harvard School of Public Health, Boston, Massachusetts, United States of America; 6 Pathogen Sequencing Unit, Wellcome Trust Sanger Institute, Hinxton, United Kingdom; 7 Department of Medicine, The University of Melbourne, Melbourne, Australia; 8 College of Osteopathic Medicine, Michigan State University, East Lansing, Michigan, United States of America; 9 Department of Basic Medical Sciences, College of Medicine, Blantyre, Malawi; Seattle Biomedical Research Institute, United States of America

## Abstract

*Plasmodium falciparum* is unique among human malarias in its ability to sequester in post-capillary venules of host organs. The main variant antigens implicated are the *P. falciparum* erythrocyte membrane protein 1 (PfEMP1), which can be divided into three major groups (A–C). Our study was a unique examination of sequestered populations of parasites for genetic background and expression of PfEMP1 groups. We collected post-mortem tissue from twenty paediatric hosts with pathologically different forms of cerebral malaria (CM1 and CM2) and parasitaemic controls (PC) to directly examine sequestered populations of parasites in the brain, heart and gut. Use of two different techniques to investigate this question produced divergent results. By quantitative PCR, group A *var* genes were upregulated in all three organs of CM2 and PC cases. In contrast, in CM1 infections displaying high levels of sequestration but negligible vascular pathology, there was high expression of group B *var*. Cloning and sequencing of *var* transcript tags from the same samples indicated a uniformly low expression of group A-like *var*. Generally, within an organ sample, 1–2 sequences were expressed at dominant levels. 23% of *var* tags were detected in multiple patients despite the *P. falciparum* infections being genetically distinct, and two tags were observed in up to seven hosts each with high expression in the brains of 3–4 patients. This study is a novel examination of the sequestered parasites responsible for fatal cerebral malaria and describes expression patterns of the major cytoadherence ligand in three organ-derived populations and three pathological states.

## Author Summary

One of the most severe forms of malarial disease is cerebral malaria, which disproportionally affects young children. In this disease, the parasite places proteins on the red blood cell surface, providing a “smokescreen” by which they evade host immunity and hide in organ blood vessels, blocking them and causing tissue damage. It is impossible to study parasites in the organs during life and autopsy studies on children with malaria are exceedingly rare. In Malawi, we examined parasites from the brain, heart and intestine of twenty cases of fatal malaria including controls with low numbers of malaria parasites but another identified cause of death. We found little difference in the category of proteins the parasites used in controls and cerebral malaria, although a small number of specific proteins were detected in multiple infections. In an alternative form of malaria in which the brain is heavily infected but shows no evidence of damage, we found a different set of proteins at high proportion. However, as these children were typically older and most were infected with HIV, we could not determine which of these factors was most important. Interactions between host and parasite have the potential to influence disease outcomes.

## Introduction

Protection from symptomatic falciparum malaria develops through successive acquisition of antibodies to variant surface antigens expressed on host erythrocytes. Severe malaria is thought to occur in part when a parasite variant exploits a gap in the host antibody repertoire. The major parasite antigen implicated in this process is the *Plasmodium falciparum* erythrocyte membrane protein 1 (PfEMP1), which is encoded by approximately 60 *var* genes per genome that are expressed in mutually exclusive fashion. This exclusivity shields most of the antigens from the immune system at any given time, contributing to the incremental accumulation of host immunity. There is, however, a suggestion that switching between genes is structured and results in certain genes being preferentially expressed [1], [2], [3]. *Var* genes are mainly clustered in sub-telomeric regions surrounded by repeat sequences, an arrangement that facilitates high rates of recombination and mutation [4].

The *var* genes encoding PfEMP1 family members are sub-divided into three major groups labeled A, B and C based on motifs in non-coding sequences and locus position, and consist of characteristic arrangements of adhesive domains [5], [6], [7]. The two major domain types are the Duffy-binding like domain (DBL) and the cysteine-rich interdomain region (CIDR). These domains are further classified into a series of cassettes with potential biologically relevant properties, particularly the DC8 and DC13 subset that adhere promiscuously to diverse endothelial cell types through endothelial protein receptor C (EPCR; [8], [9], [10]). The EPCR-binding *var* are classified as group A (DC13 and others) or an A/B chimera (DC8) *var* [9]. These domains are located in the extracellular portion of PfEMP1 and are displayed on the surface of the parasitised red blood cell (pRBC) where they bind to endothelial receptors on host microvasculature, a process known as cytoadherence. This process removes pRBC from the circulation, leading to sequestering of pRBC in post-capillary venules where they thrive in a hypoxic environment and avoid splenic clearance. In large accumulation, sequestration can lead to blockage of blood vessels, haemorrhage and formation of thrombi and, in some cases, migration of monocytes to the point of vascular damage, all of these processes being commonly identifiable in the pathology of paediatric cerebral malaria (CM) [11].

A number of specific binding interactions between endothelial receptors and PfEMP1 adhesive domains are well established, such as CIDRα domains adhering to CD36, the DBLβ domain binding to intercellular adhesion molecule-1 (ICAM-1) [12], [13], [14], and domain subtypes CIDRα1.1 of DC8-containing PfEMP1 variants and CIDRα1.4 in DC13 genes with EPCR [9]. Other potential binding interactions are less well understood although a range of parasite and host receptors may support pRBC adhesion. The most striking example is the adherence of a PfEMP1 encoded by the group E gene, *var2csa*, to chondroitin sulphate A on syncytiotrophoblasts in the placenta [15]. The unique nature of this interaction, restricted to a single organ, raises hopes of using this *var* gene in a vaccine specifically targeting malaria in pregnancy.

The tissue pathology of paediatric cerebral malaria is, by its very nature, inaccessible to direct study and therefore poorly understood. Studies on circulating parasites have demonstrated that there is high turnover of the antigens expressed during disease and in asymptomatic infections [16], [17], [18] and that severe disease syndromes are often associated with higher expression of group A *var* genes in peripheral blood, albeit inconsistently [19], [20], [21], [22], [23], [24]. It has been suggested that two subsets of group A *var* are responsible for differing disease manifestations, with one being associated with respiratory distress and the other with impaired consciousness [25].

Our work is part of a paediatric malaria clinical and pathological study in Malawi in which more than 100 children with fatal encephalopathy, including 73 with clinically-defined cerebral malaria, have been studied between 1996 and 2011. For the present study, we have divided the cases into three diagnostic groups based on their clinical, autopsy and histological features: CM1, clinically defined CM with cerebral sequestration in the brain but with no associated vascular pathology; CM2, clinically defined CM with cerebral sequestration and intra- and peri-vascular pathology such as ring haemorrhages, thrombi and infiltration of monocytes; and a third group of parasitaemic controls (PC) made up of comatose patients with circulating *P. falciparum* parasitaemia but minimal sequestration, and a non-malarial cause of death. This unique study allows us to access sequestered populations of pRBC to investigate the features of parasites located in organ microvasculature and to identify the ligands that may be implicated in cerebral cytoadherence.

Owing to the high diversity of *var* antigens and, therefore, unknown contingent of *var* genes present in clinical *P. falciparum* isolates, the methodology for studying their expression in clinical samples requires the use of degenerate primers. Previous clinical studies exclusively used peripheral blood samples and one of two methods: amplification of *var* transcripts using “universal” primers that amplify the vast majority of *var* genes, or more recently, quantitative real time PCR (qRT-PCR) targeting *var* groups A, B or C [19], [20], [21], [22], [23], [24]. We used both techniques in order to compare *var* group expression in these organ-based parasite populations with contemporary qRT-PCR studies, and also for the more rich detail supplied by cloning and sequencing of individual *var* tags. Our results demonstrate why these methods have not previously been presented simultaneously using clinical isolates as their results are starkly contradictory. We present both data sets here with a discussion of the pros and cons of each approach to allow readers to make their own judgment on the relative merits of each.

## Results

### Patient selection

Twenty patients were selected from the paediatric malaria study. Their details are in Table 1. At the time of selection, 87 cases of fatal malaria and controls were available. We chose the patients based on diagnostic classification [26], time between death and post-mortem examination, storage conditions of the samples and from data extracted from our pilot study on *var* expression [27]. This had raised the possibility of limited diversity of *var* genes within a malaria season and so the patients were chosen at a fixed ratio over five malaria seasons. Among autopsy-confirmed CM cases, it was previously shown that approximately two thirds are CM2 and one third CM1 [26], and therefore we sampled cases at a ratio of 2 CM2: 1 CM1: 1 PC. One PC case was later revised as CM2 based on final histological examination, leaving us with a final selection of 11 CM2: 5 CM1: 4 PC.

**Table 1:** Clinical details of patients.

Diagnosis	Case no.	Year of admission	Age^a^	HIV status	Time to death^b^	Admission parasitaemia^c^	Final parasitaemia^c^
*CEREBRAL MALARIA*							
CM2	28	1999	61	-	00:30	424,000	424,000
	34	1999	70	-	02:45	74,319	74,319
	61	2002	26	-	04:15	42,027	42,027
	62	2002	10	-	01:10	1,056,607	1,056,607
	63	2002	79	-	28:10	8,212	400
	64	2002	60	+	07:00	28,842	28,842
	68	2003	156	+	15:25	20,080	44,680
	75	2003	144	+	07:50	215,300	36,250
	78	2003	15	-	02:00	637,000	637,000
	82	2004	31	-	56:30	197,820	0
	83	2004	26	-	13:45	22,000	6,000
*mean*			*61.6*		*12:40*	*247,837*	*213,648*
*st dev*			*49.4*		*16:42*	*334,794*	*348,042*
CM1	37	1999	6	+	38:00	616,400	10,000
	38	1999	84	-	35:40	782,320	5,474
	74	2003	103	+	05:25	204,000	204,000
	79	2003	79	+	04:40	34,400	34,400
	84	2004	106	+	21:07	201,829	834
*mean*			*75.6*		*20:58*	*367,790*	*50,942*
*st dev*			*40.6*		*15:55*	*315,896*	*86,537*
*PARASITAEMIC CONTROL*							
PC							
d	31	1999	39	+	22:00	159,434	188,432
d	45	2000	28	-	23:25	100,519	197
e	77	2003	7	-	04:05	66,844	66,844
f	80	2004	63	-	12:00	23,500	63,200
*mean*			*34.3*		*15:22*	*87,574*	*79,668*
*st dev*			*23.3*		*09:05*	*57,349*	*78,700*

a months,

b hours:minutes,

c parasites/l in peripheral blood,

d pneumonia (Streptococcus),

e malaria parasitaemic with non-malarial cause of death,

f meningoencephalitis.

### Restricted genetic diversity of CM patients

Sixteen of the twenty patients were assessed for *P. falciparum* genetic diversity in the brain, heart and gut by *msp* typing and barcoding (S1 Figure). Four patients were excluded because the samples had low *P. falciparum* DNA concentration or failed extraction. *msp2* typing provides the multiplicity of infection (MOI) with non-quantitative detection of all genetic variants [28]. Barcoding assigns a unique identifier to each infection and determines whether one of the variants is present at disproportionately high levels and dominates the infection (monoallelic) or whether it is a heterogeneously mixed infection (multiallelic) [29], [30].

All of the infections were genetically distinct (S1 Figure). There was a mean of 2.4±0.7 genetic variants per patient, and between 1.5±1.1 and 2.2±1.1 variants per organ, comparable to contemporary studies in Malawi [31], [32]. There were no significant differences in MOI between organ types, diagnostic groups or season. We also did not observe any correlation between genetic diversity and the density of peripheral parasitaemia.

The proportion of monoallelic infections were 50% for CM2, 40% for CM1 (although patient 37 had only a single heterozygous call) and 33% for PC cases. Three CM2 cases that were classified as monoallelic by barcoding were shown to have a second genetic variant by *msp*2 typing (patients 28, 68 and 78 in S1 Figure). This is explained by the higher sensitivity of the *msp* technique, which is not quantitative and detects any amount of DNA, whereas barcoding can overlook variants present at low levels (<5% of total parasitaemia) [29].

### Proportion of *var* gene groups in patient isolates is similar to reference genome 3D7

As a precursor to expression analysis, the twenty malaria cases were investigated to determine the proportion of *var* gene groups in the genomes of the infecting *P. falciparum* isolates to confirm that the expected ratio of A:B:C genes is comparable to the 3D7 reference genome and other sequenced genomes [5], [33]. We quantitatively analysed *var* gene groups A, B and C by qPCR of genomic DNA from tissue biopsies. The genomic distribution of the three *var* groups indicated similar distributions in all diagnostic categories and between organs.

### Group A genes are highly expressed in organ microvasculature

**Figure 1:**
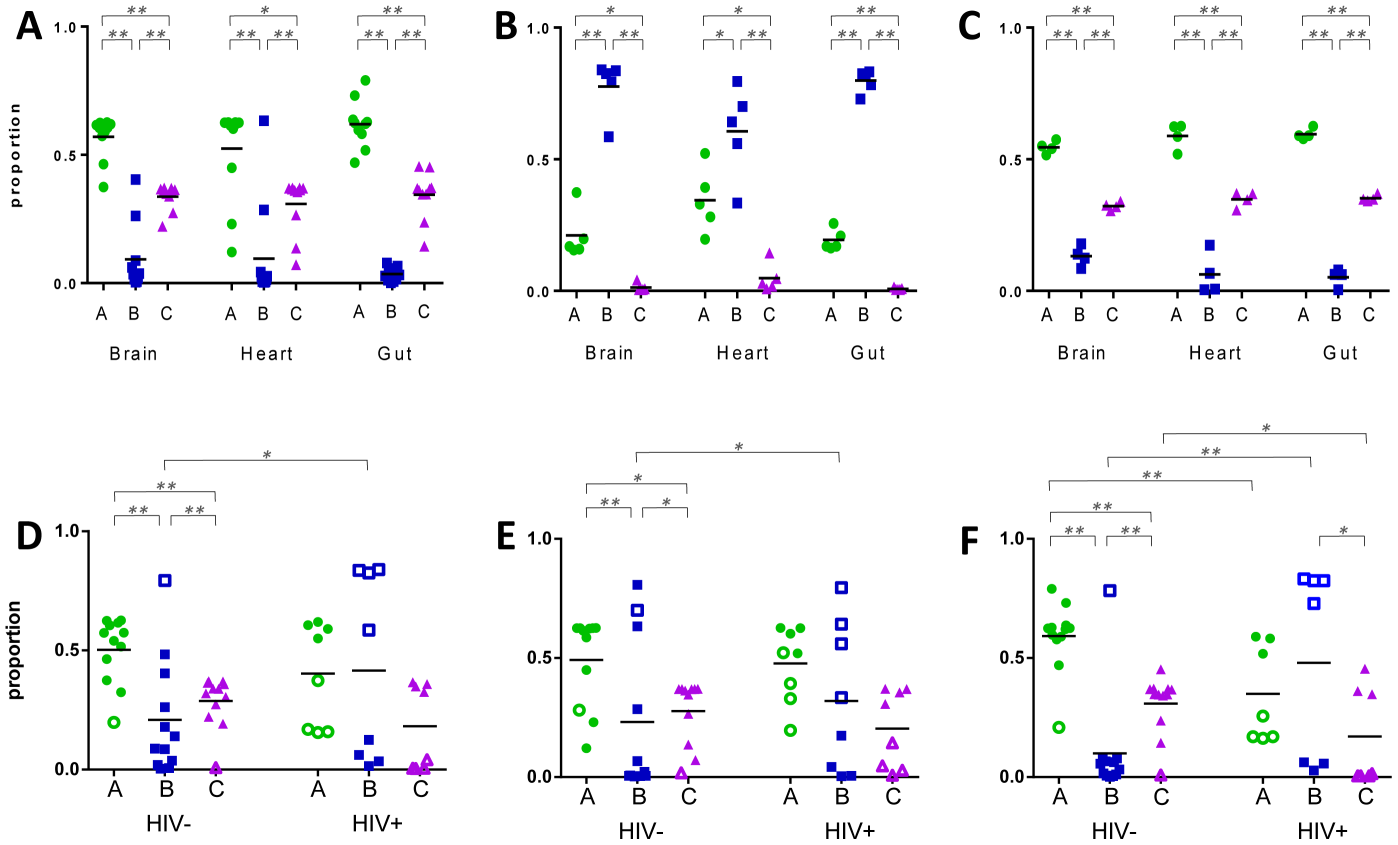
Expression of *var* gene groups in the organs of paediatric hosts. Primers specific for *var* groups A, B and C were used to measure their relative expression in tissue biopsies from fatal paediatric malaria patients. Panels A–C display hosts within diagnostic groups CM2 (A), CM1 (B) and parasitaemic controls (C). Panels D–F represent *P. falciparum* populations in the brain (D), heart (E) and gut (F) of HIV-infected (HIV+) and uninfected (HIV-) hosts. Each dot point represents analysis from a single organ biopsy from one patient and the horizontal lines depict the mean level of expression for each group. In panels D–F, CM2/PC hosts are denoted by filled shapes and CM1 patients with open shapes. * p<0.05, ** p<0.005.

We used qRT-PCR to determine the expression levels of *var* gene groups A, B and C in the infecting *P. falciparum* isolates. All samples included in the analysis showed no significant differences in observed raw C_T_ values between clinical groups. Only three samples; patient 62 heart, patient 75 gut and patient 78 brain were excluded from the analysis due to failure of extraction or amplification of cDNA. In CM2, group A *var* genes were expressed at high levels in the brain compared to group B and C genes (p = 0.001, ANOVA Bonferroni; Fig. 1A), consistent with peripheral blood studies linking group A expression with severe forms of disease [19], [21], [23], [24]. Group A genes were highly expressed in all three organs and group B expression was lowest (p = 0.001). The same pattern of expression was observed in PC cases (Fig. 1C), despite their having a lower mean density of peripheral parasitaemia at both admission and death compared to CM2 patients, and their lack of the pathology characteristic of severe malaria (Table 1).

In contrast, CM1 patients had a unique pattern of expression of the three *var* antigen groups (Fig. 1B), with group B *var* being the most highly expressed and group C being the least expressed, regardless of organ (p = 0.001).

### Potential influence of HIV infection on pRBC sequestration

We next investigated whether the disparate *P. falciparum* antigen expression in CM1 infections might be associated with unique host characteristics. CM1 accounted for a third of confirmed CM cases in the clinicopathology study and could only be identified definitively by post-mortem examination. The CM1 group did not significantly vary in other patient characteristics outlined in Table 1 (age, time to death, admission or final parasitaemia). However, CM1 patients were more likely to be HIV+ than CM2 (p = 0.049; Kruskal-Wallis test) and we therefore re-analysed the data taking this into account. Owing to the small numbers of patients, we divided them into HIV-infected (HIV+) and uninfected (HIV-), irrespective of malaria diagnosis.

Patient distribution was respective of their diagnostic group and in each organ the *var* expression pattern in HIV- cases closely resembled that of CM2 and PC, with high group A expression and lowest expression of group B genes (Fig. 1D–F). A different pattern was seen in HIV+ patients, with high and low expressing populations for each *var* group. Group B genes were significantly over-expressed in HIV+ cases compared to HIV- in all three organs (brain: p = 0.040; heart: p = 0.035 and gut: p = 0.039, ANOVA Bonferroni).

Age is a potential confounder in this result; the HIV+ patients were significantly older than HIV- (mean of 86.6 and 41.7 months, respectively; p = 0.020). However, linear regression modeling found no association between *var* expression levels and either age, diagnosis or HIV status. The small sample size limits the power of statistical analyses and we do not have indicators of the stage of HIV disease in these patients who presumably span a range of immunological deficiency.

The significant differences in *var* expression between HIV+ and HIV- patients suggest a potential influence of HIV infection on falciparum malaria but the relative influence of HIV, age and the CM1 condition itself cannot be thoroughly assessed in our sample population. An analysis of the impact of HIV on malaria pathology is underway in the larger clinicohistopathology study of which our patients are a subset.

### Organ-derived pRBC populations express dominant *var* genes

Expression of *var* transcripts in post-mortem organ samples was further investigated by cloning and sequencing of DBL1α transcripts. We will refer to each sequenced product as a “tag” and to each DBL1α sequence variant as a “type”. From our pilot study [27], we calculated that 100 tags were sufficient to characterise *var* antigen expression within an organ sample. We attempted to clone 100 tags, in two batches of 50, from each of brain, heart and gut biopsies from 20 patients. Of these, 58 biopsies were successfully processed from which we cloned a total of 5800 tags, 5153 of which were identified as *var*-DBL1α sequence.

**Figure 2:**
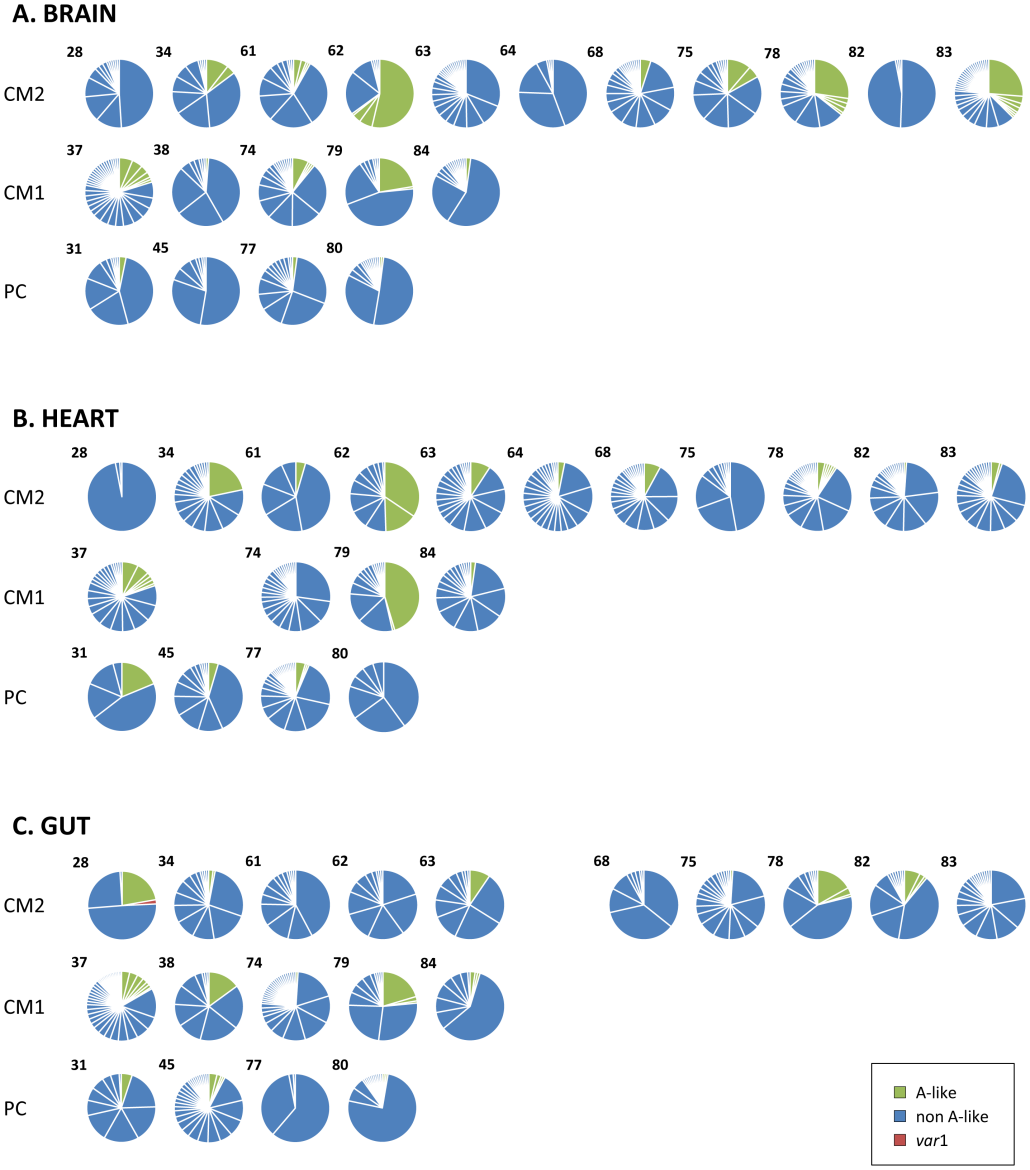
Distribution of individual *var*/PfEMP1-DBL1α types in fatal paediatric malaria hosts. 100 DBL1α tags were amplified and sequenced from each tissue biopsy and different sequence variants identified. Each pie graph represents all DBL1α types from a single organ of an individual host shown in the brain (A), heart (B) and gut (C). Case numbers are shown in the upper left corner of each graph and they are arranged by diagnostic group (CM, cerebral malaria; PC, parasitaemic controls). Tags are coloured by whether they are classified as group A-like *var* types (green) or non-group A (blue).

Tags were considered the same if they possessed >95% nucleotide identity, which gave 613 discrete sequence types (S1 Figure). 218 (35.6%) of *var* types were unique, cloned only once from a single organ biopsy. This accounted for 4% of all tags sequenced. Approximately a quarter of types (163; 26.6%), or 60% of tags, were expressed in multiple organs of the same patient. We found a mean of 39±17 DBL1α types per patient, and 17±10 per organ (Fig. 2). There were no significant differences in the number of unique sequence types identified between organs or diagnostic groups.

**Figure 3:**
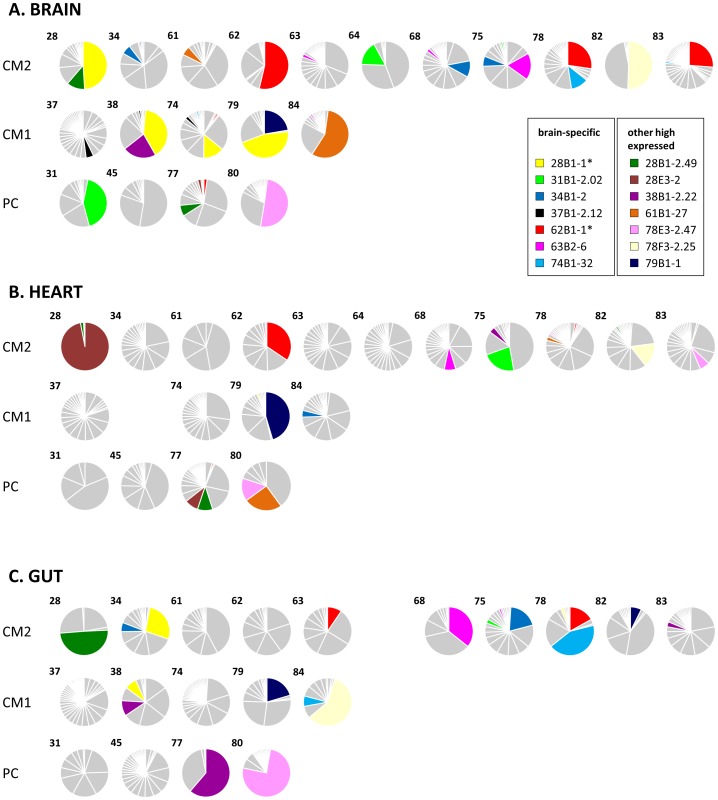
Distribution of individual *var*/PfEMP1-DBL1α types in the organs of fatal paediatric malaria hosts. Each pie graph represents all DBL1α variants from a single organ of an individual host shown in the brain (A), heart (B) and gut (C). Case numbers are shown in the upper left corner of each graph and they are arranged by diagnostic group (CM, cerebral malaria; PC, parasitaemic controls). These charts are identical to those in Fig. 2 except that sections are shaded to highlight the DBL1α types that were detected in the highest number of different hosts. Further information on these can be found in S1 Table. The two DBL1α types detailed in results are marked with an asterisk.

*P. falciparum* infections analysed from peripheral blood and *in vitro* have been shown to express a predominant *var* gene at population level, and the same was observed in these organ-derived samples [2], [22], [34], [35]. In our current analysis, dominance of expression was defined as a sample from which at least 33% of tags cloned from a single biopsy sample were identical, so that no more than two sequences could be dominant in any one sample (in theory this would be three, but we did not find so few sequences from a single sample). Amongst our patients, 12 (60%) had a dominant *var* type expressed in the brain, compared to 7 (35%) in the heart and 8 (40%) in the gut (Fig. 2). It was rare to find the same *var* type expressed at dominant levels in more than one organ; this only occurred in only two patients (Fig. 3; types 62B1-1 and 78E3-2.47).

### Identical *var* transcripts detected in brain microvasculature of multiple patients

The patients recruited into this study live in an area of high malaria transmission with a complex degree of genetic variability in the infecting isolates, and each of the 20 infections was genetically distinct (S1 Figure and [30]). Such variability, and the very low frequencies at which identical DBL1α types are observed in multiple patients in peripheral blood studies [19], [36], [37], originally led us to expect distinct *var*/PfEMP1 populations in the 20 patients. However, our pilot study demonstrated considerable overlap between DBL1α types expressed in a small number of patients from a single malaria season [27]. Moreover, their organ localisation was largely conserved. We therefore further investigated these phenomena in this larger study.

140 (22.8%) of the 613 unique DBL1α types identified were expressed in multiple patients (S2 Figure). When we considered the number of times each type was detected, 57.5% of tags were detected in more than one host, suggesting that DBL1α types with high expression levels are more likely to be detected in multiple patients. Individual *var* types were observed in up to 7 different patients, and 37 (6%) of types were detected in at least three hosts (Fig. 3).

15.0% of DBL1α types were detected in the same organ of multiple patients (Fig. 3 and S2 Figure). 37.2% of *var* types detected in the brain of one host were also detected in the brain of another patient, with non-significantly lower overlap in the heart and gut (32.4 and 28.2%, respectively). Those types observed at higher frequency in any one sample were more likely to be detected in others, such that 734 (41.1%) of all brain-derived DBL1α tags were detected in multiple brain samples, whereas overlap was 24.0% in the heart and 31.26% in gut biopsies (p<0.005 for both).

These findings contrast with peripheral blood studies in which fewer than 5% of identical DBL1α types are typically detected in multiple patients, apart from in regions of low transmission [19], [36], [37]. We compared our own peripheral blood samples from a related study in which 17–100 DBL1α cDNA tags were amplified and sequenced from each of 15 paediatric malaria patients (S2 Figure). We observed that 17 (8.9%) of the total 191 DBL1α types were shared between patients, accounting for 26.1% of all tags sequenced. Two patients had 9 of these shared types, accounting for much of this overlap (S3B Figure, patients 92 and 95). This level of overlap is more in agreement with previous peripheral blood studies, and suggests that there is a restricted number of *var*/PfEMP1 variants expressed by sequestered pRBCs in the microvasculature of the brain, gut and heart, compared to those expressed in circulating populations.

We did not observe any associations between the number of shared DBL1α types in organ samples, diagnostic group or seasonality. In contrast to our pilot study, we observed no restricted expression of particular *var* sequences within a malaria season.

### Commonly detected *var* antigenic types

The *var* tags cannot be classified by A–C groups due to their incomplete sequence of the DBL1α domain. To further investigate the potential role of group A *var* genes in severe malaria, we identified group A-like antigens by their possession of at least one polymorphic sequence block drawn from a defined subset called block-sharing group 1 and the presence of two conserved cysteine residues, rather than the more common four. This was performed using an analysis method kindly provided by Pete Bull and described previously [25].

The majority (91.9%) of DBL1α types were of the non-A-type, and only 8.8–11.9% of types were A-like in each organ (Fig. 2). This is in contrast to the qRT-PCR results in which more than half of CM2 and PC transcripts were group A (Fig. 1). When the frequency of detection was taken into account the gut had only 7.9% of tags that were A-like, significantly less than the brain or heart (12.6 and 11.9% respectively, p<0.005 for both). Isolates from the peripheral blood study had a higher proportion of A-like sequences (14.3%; S3A Figure).

Two DBL1α types were frequently detected in brain biopsies (Fig. 3, starred). *var*62B1-1 (accession number KC678324) is A-like, comprises a DBL1α_1_ domain and was the most commonly detected type, being observed in seven hosts. In three CM2 patients: 62, 78 and 83, it was the mostly highly expressed *var* type in the brain. It was also amplified in two additional brain samples and in three samples each from heart and gut (see S2 Table for details). A nearly identical sequence was previously detected in Kilifi, Kenya, but this sequence had no particular distinguishing characteristics in that study (S1 Table; [38]). Downstream sequencing revealed a DBL1α1.1-CIDR1α1.7-DBL2β3 structure (accession number KC678109).

The other tag, *var*28B1-1 (accession number KC678110), was also the dominant *var* antigen in three brain samples, and was the second most expressed type in the brain of a fourth patient (Fig. 3, starred). Interestingly, 4 of the 6 patients in which this type was detected had a CM1 diagnosis. A highly similar sequence (97% identity) was also detected in two severe malaria patients in Kilifi, Kenya, where it was strongly associated with high rosetting rates and may be disproportionally prevalent in East African isolates (S1 Table, [19]).

## Discussion

CM is one of the most dangerous and often fatal complications of *P. falciparum* infection in African children. Using the unique resources of the clinicopathological study of paediatric malaria in Blantyre, Malawi, we have analysed sequestered populations of pRBC in the brain, heart and gut of 20 cases of fatal malaria. These patients hosted genetically unique parasite populations with limited variation in their distribution between organ sites within individual CM cases.

High expression of group A *var* has been linked to severe and cerebral malaria *in vitro* and in peripheral blood samples from *P. falciparum-*infected children [19], [21], [23], [24], [39]. Using qRT-PCR targeting *var* groups A, B or C, we also found group A *var* expressed at high levels in organ-derived parasite populations. Group A *var* were upregulated in both CM2 cases and parasitaemic controls. This suggests that group A antigens promote higher rates of sequestration irrespective of the severity of symptoms. Our third diagnostic category was CM1 in which patients carried a heavy sequestered load of pRBC in the brain with limited vascular pathology. In these patients, upregulation of group B antigens was observed (Fig. 1). Transcription of group B *var* has been associated with severe symptoms in a minority of peripheral blood studies although there was no ability to distinguish between CM1 and CM2 diagnoses in these sites [20], [23], [40].

Previous clinical studies using the cloning and sequencing approach have also sometimes found expression of group A-like *var* associated with severe malaria, although the limited sequence information contained in these tags has hindered further classification of these antigens [17], [18], [19], [25], [41], [42]. In contrast to this, and to our own qRT-PCR results, group A *var* constituted no more than 12% of *var* transcripts expressed by our organ-derived parasite populations (Fig. 2). Using this method, equivalent proportions of A-like *var* were expressed by parasites in CM1, CM2 and PC patients.

There are clear disparities between the level of A-like *var* expression measured using qRT-PCR and cloning methods. The question is which of the two is the more reliable. Gatton *et al* found good concordance between the two techniques using *P. falciparum* isolate 3D7, and in a Ugandan study the same highly expressed genes were detected by both methods in two laboratory isolates and three of five clinical isolates [43], [44]. However, both of these studies used gene-specific primers for the qRT-PCR, not degenerate primers as used in this study. In addition, they were working with laboratory-adapted isolates whereas we were using tissue samples in which *P. falciparum* genetic material constitutes only a fraction of the total. This pushes both techniques to the limits of their capacity. The use of degenerate primers risks amplification of contaminating human DNA although any reactions with multiple products visible by dissociation curve were rejected.

The qRT-PCR data were consistent in multiple independent replicates and cases for which this was not true were excluded. The primers are not fully efficient for the myriad varieties of *var* genes and may subsequently exhibit bias, especially in clinical isolates with unknown *var* repertoires [20]. In order to ensure that the genomic composition of *var* groups was similar to the reference genome 3D7 and to highlight potential primer bias, we quantified the ratio of *var* groups A, B, and C in the genomic DNA of all twenty malaria cases by qRT-PCR and found no difference in their relative proportions compared to 3D7. Differences we observed in *var* group expression were therefore likely due to transcriptional regulation and not primer bias. qRT-PCR preferably uses probes specific for each *var* gene group [20]. However, it was extremely difficult to get a consistent product using this technique on our post-mortem samples, presumably due to high levels of human DNA. As more *var* genes are sequenced from geographically diverse populations the design of degenerate probes specific for each *var* group will improve.

The cloning and sequencing approach should be considered semi-quantitative; particularly in this case where, because of the low proportion of *P. falciparum* DNA in tissue samples, two rounds of amplification were required. While interpreting the proportion of each *var* tag with some caution, the data consistently showed that *var* expression in individual organs tends to be dominated by a limited number of sequences, and commonly by one *var*/PfEMP1 type, particularly in the brain (Fig. 3). This is in accordance with our pilot study and is also observed in circulating populations [17], [18], [19], [25], [27], [41], [42]. Rarely was the same DBL1α tag the dominant transcript in different organs of a single host.

Conversely, there was considerable overlap in *var* types expressed in multiple hosts, often with organ localisation conserved, even though the infecting isolates were genetically distinct. Commonly observed *var*-DBL1α types may be partially explained by recent modeling data showing a positive correlation between transmission intensity and overlap of *var* repertoire, independent of MOI [45]. Peripheral blood studies (including our own) show low rates of identical *var* types being expressed in multiple infections, which may imply that a restricted number of *var* antigens are used by sequestering *P. falciparum* parasites in the brain, heart and gut. Furthermore, we identified two *var* tags that were expressed at high levels in the brains of 3–4 patients, and in all organs each tag was detected in 6–7 individual hosts (S2 Table). This is a remarkable finding given the distinct genetic barcodes of each infection. In areas of high transmission, severe malaria typically affects those in the earliest years of life, but only a minority of children develop severe disease syndromes. This suggests that a limited set of parasites are capable of causing severe disease before protective immunity is achieved [46], [47]. This could be due to a restricted antigenic repertoire with efficient or diverse adhesive capabilities that are preferentially transcribed either through hierarchical expression, switching at rapid on-rates or slow off-rates, or both [1], [2].

The overall lack of organ specificity of any *var* group is in agreement with *in vitro* studies showing that pRBC selected on cerebral endothelia, or expressing group A and A/B PfEMP1 containing DC13- or DC8 cassettes, promote cytoadherence across a range of organ-derived endothelial cells [10], [48]. The receptor for pan-endothelial adhesion, EPCR, is not detectable in the cerebral microvasculature of Malawian children who have died of malaria but its critical role in pathogenesis may be earlier [49]. The many studies linking high group A *var* expression in circulating *P. falciparum* to severe symptoms suggest that there must be high sequestration of group A-expressing pRBC at the early symptomatic stage of disease when these patients would be recruited [17], [18], [19], [25], [41], [42].

This study demonstrates the inherent difficulties in examining parasite expression in post-mortem samples but has nevertheless illustrated that at this late stage of disease, similar proportions of *var* gene transcripts are found in the brain, heart and gut of paediatric malaria patients although the individual genes transcribed differ. High expression of group B *var* is found in the less common CM1 diagnostic group but the skewed characteristics of these hosts do not allow us to distinguish between cause or effect. In future, it would be interesting to investigate the frequency of *var* domain subclasses or DCs if the considerable technical difficulties of working with post-mortem samples can be overcome [8].

## Materials and Methods

### Patient selection

Patients were admitted to the Paediatric Research Ward (PRW) at the Queen Elizabeth Central Hospital (QECH), Blantyre, Malawi, prior to their death. The clinical case definition for CM is a Blantyre Coma Score of ≤2, peripheral *P. falciparum* parasitaemia, and no other identifiable cause of coma. Anaemic children (haematocrit <15% at any time during hospitalisation) were excluded from the study. Treatment that was prescribed to each patient was as previously described [26]. The time between admission and death varied between 30 minutes and 2.3 days with a mean of 15∶17 hours, and autopsies were performed a median of 8±5.5 hours following death. Following post-mortem examination, malaria cases were divided into three diagnostic groups described above and more extensively elsewhere [26]. Details of these patients are in Table 1.

Patients for the peripheral blood analysis were recruited from the Paediatric Research Ward at Queen Elizabeth Central Hospital, Malawi. They were identified by fever of >37°C or history of fever in last 24 hours and *P. falciparum* asexual parasitaemia of any density observed by light microscopy.

### Nucleic acid extraction and processing

Approximately 0.4×0.4×1 cm tissue samples were collected at autopsy, submerged in RNAlater (Qiagen, UK) and snap frozen in liquid nitrogen. At extraction, samples were thawed on ice then ground to powder using a liquid nitrogen-cooled mortar and pestle. Half was put in 4 ml of DNA extraction buffer (10 mM Tris-HCl, 0.1 M EDTA, 0.5% sodium dodecyl sulphate, 20 µg/ml RNase A, pH 8.0) and half in 4 ml of Trizol (Sigma Aldrich, UK) for RNA extraction.

For isolation of genomic DNA, samples were incubated at 37°C for 60 minutes (min) and proteinase K added to a final concentration of 0.1 mg/ml. Samples were incubated at 50°C for 3 hours with regular mixing by gentle inversion. After cooling to room temperature (RT), 5 ml of phenol:chloroform:isoamyl alcohol (25∶24∶1; PCI) was added and samples were placed on a rotary mixer for 10 min. After centrifugation at 5,000 g for 30 min, the aqueous phase was carefully transferred to a fresh tube. If there was still a lot of material at the interface or some of this material was carried over with the supernatant a second PCI extraction was performed. Subsequently, 1× volume of chloroform was added and after 10 min on a rotary mixer the samples were centrifuged at 5,000 g for 30 min and the aqueous phase transferred to a fresh tube. DNA was precipitated with addition of 0.2× volume of 10 M ammonium acetate and 2 volumes of ethanol. Precipitated DNA was transferred using a disposable sterile loop to a fresh tube containing 1 ml of 70% ethanol, washed by inversion and pelleted at 5,000 g for 10 min. The ethanol was aspirated and the wash step repeated. The pellets were air dried and resuspended in 0.2–1 ml of 10 mM Tris-HCl, 1 mM EDTA, pH 8.0 depending on the pellet size, resuspended by overnight incubation at RT and stored at 4°C.

For extraction of RNA, samples in Trizol were centrifuged at 12,000 g at 4°C to pellet insoluble material and the supernatant transferred to a new tube. After 5 min incubation at RT, a 0.2× volume of chloroform was added and the tubes shaken for 15 seconds (sec). Following 3 min incubation at RT, the samples were centrifuged at 12,000 g for 30 min at 4°C. The aqueous phase was transferred to four 1.5 ml tubes and 0.5 ml of isopropanol added to each. The RNA was precipitated by overnight incubation at 4°C followed by centrifugation at 12,000 g for 30 min at 4°C. The pellets were each washed in 1 ml of 75% ethanol, centrifuged at 7,500 g for 5 min at 4°C then aspirated and air dried. The pellets were each resuspended in 20 µl of RNA storage solution (Life Technologies, UK) and stored at −80°C. This method is adapted from Kyes *et al.* [50].

5–10 µl aliquots of RNA were treated with DNase I to remove contaminating genomic DNA using the DNA-free RNA kit (Cambridge Bioscience, UK) according to manufacturer's instructions. Removal of DNA was confirmed by performing two rounds of *var* universal PCR as described below (with no reverse transcription step). 1 µl of RNA was also assessed using a NanoDrop (Nanodrop, UK) and degraded samples were discarded. cDNA was synthesised using the Retroscript kit (Life Technologies, UK); briefly, 2 µl of DNA-free RNA was mixed with 50 pmol of random decamers and 8 µl of nuclease-free water and incubated at 65°C for 5 min. Samples were briefly centrifuged then placed on ice for 5 min. To each sample was added 2 µl of RT buffer (0.5 M Tris-HCl, pH 8.3, 0.75 M KCl, 30 mM MgCl_2_, 50 mM DTT), 30 pmol of deoxynucleotide triphosphates (dNTPs), 10 units of RNase inhibitor and 100 units of MMLV-reverse transcriptase. cDNA was synthesised at 42°C for 60 min and then the reverse transcriptase was inactivated at 92°C for 10 min. The same cDNA samples were used for both qRT-PCR and *var* amplification and cloning.

*P. falciparum*-infected peripheral blood samples were subjected to a pre-lysis step whereby 5 µl of 10% saponin was added per 200 µl of blood, vortexed vigorously and then centrifuged at 6,000 g for 5 min. The supernatant was discarded and the pellet was resuspended in 0.2 ml of 1× phosphate buffered saline (pH 7.2–7.4; PBS), at which point the samples were processed using the QiaAmp Blood Miniprep Kit (Qiagen, UK) according to manufacturer's instructions.

### Nucleic acid amplification

Typing of *msp*2 was performed as described in Snounou *et al.* [28]. DNA quantification and barcoding was performed using the protocol of Daniels *et al.* [29] using SYBR green detection for quantification and Taqman for barcoding (Applied Biosystems, UK). All parasite DNA concentrations were standardised to 0.001 ng/µl before PCR analysis.

For differential *var* group transcriptional analysis, quantitative real-time polymerase chain reaction (qRT-PCR) specific for all 3 *var* gene groups using a method modified from Kaestli *et al.* [20]. Prior to qRT-PCR, 1 µL of 0.001 ng/µl cDNA or gDNA samples were amplified in a primary PCR on a Veriti thermocycler model 9902 (Applied Biosystems, UK). A 50 µl reaction containing 2 mM of MgCl_2_, 0.4 M of dNTPs, 0.25 units of *Taq* DNA polymerase (Invitrogen, UK) and 400 nM of each primer was amplified using an initial incubation of 94°C for 5 min followed by 14 cycles for gDNA and 16 cycles for cDNA of the following conditions: 95°C for 30 sec, 52°C for 1 min and 64°C for 70 sec in 50 µl volume. The forward primer for each *var* group was A: 5′-AACTTACCATAAATTATCATCAAA, B: 5′-CTCATWTATAATTTTASAAAATAWAWAAAAC, C: 5′-AATATTCATATTCCCACATTRTCATATAT and reverse primer DBL1αrev: 5′-CCWATRKCDGCAAAACTBCKWGC. The primary PCR product was checked on 1% agarose gel electrophoresis and lack of a visible band indicated that the subsequent RT-PCR would not exceed the linear range.

qRT-PCR was performed on a 7900HT Fast Real-Time PCR System (Applied Biosystems) using primers targeting group A, B or C *var* genes designed from alignments of 5′ untranslated region *var* sequences. These reactions used 2 µl of primary product in 10 µl volume containing 1× SYBR Green and a final primer concentration of 900 nmol/L using forward primers described above and reverse primers A: 5′-TCACCTACAACAAATRTAATAAA, B: 5′-TTAWGGGAGTATWGTDATATGGTAGAAT and C: 5′-ATTATGTGGTAATATCATGTAATGG. An initial incubation of 94°C for 5 min was followed by 40 cycles of 95°C for 30 sec, 54°C for 1 min and 64°C for 70 sec.

The samples were divided by diagnosis in 384-well plates using 3D7 DNA as a positive control. All DNA samples were run in triplicate and were included in the analysis if the cycle-threshold (C_T_) was within the linear range between 15 and 31 and a product dissociation curve with a melting temperature difference of <1°C. Each plate that did not meet the above stated standards was discarded and the samples were repeated from the cDNA. Standard curves were linear over a 6-phase dilution series of 3D7 gDNA ranging between 0.8–0.00008 ng/µl, each in triplicate. The PCR efficiency (E) was calculated using the formula E = 10(1/-slope)-1. The slope was analysed close to −3.47 as recommended by the manufacturer (Applied Biosystems) to maintain maximum efficiency. The mean efficiencies of three independent standard curves with high reproducibility were 98% for var group A, 87% for var group B, and 96% for var group C.

The Kaestli *et al.* method was used to convert raw *C*_T_ values into approximate copy numbers using the formula C/E^ΔC^_T_, where *C* is the number of gene copies in *var* groups A, B, or C in the genome of the plate calibrator (3D7); *E* is the amplification efficiency of the corresponding primer pair; and Δ*C*_T_ is the difference in average *C*_T_ values between the sample and the corresponding *var* group using 3D7 DNA [20]. Finally, *var* transcript abundance was expressed as a proportion of total transcript of all *var* groups per sample.

In order to ensure that the genomic composition of the *var* subgroups was similar to the 3D7 reference genome and other sequenced genomes, the ratio of *var* gene groups A, B and C in genomic DNA of the twenty malaria cases were also quantified by qRT-PCR. As expected, the genomic distribution of the three *var* subgroups was similar among hosts from different clinical diagnostic groups as well as between the brain, heart and gut, with 7% of the overall genes amplified belonging to *var* group A, 76% to *var* group B and 16% to *var* group C.

### Amplification and sequencing of individual *var* sequences

Amplification of DBL1α tags used a nested PCR approach of Duffy *et al.* [51]. The first round utilised 2 µl of cDNA and *var*-DBL1α primers 5′-GGIGCITGYGCICCRTWYMG and 5′- TCTTCIGYCCATTCCTCGAACCA with a final concentration of 4 mM of MgCl_2_, 0.2 M of dNTPs, 1 mM of each primer and 0.25 units of *Taq* DNA polymerase (Invitrogen, UK). Following an initial incubation of 95°C for 3 min there were 50 cycles of 93°C for 30 sec, 55°C for 30 sec and 72°C for 1 min, with a final extension at 72°C for 7 min. In the second round, primers 5′-GCACGMAGTTTYGCNGATATAGG and 5′-ARATAYTGNGGSACRTARTCNARAT were used under the same conditions excepting an extension temperature of 52°C and 3.8 mM MgCl_2_. PCR products were cloned into the pGEM-T-Easy Vector System I (Promega, UK) according to manufacturer's instructions. Plasmids were extracted using the PureYield Plasmid Miniprep System (Promega, UK) as recommended by the manufacturer.

Long range PCR on *var*62B1-1 (GenBank accession number KC678324) using specific internal forward primer 5′-AGAAACGATTGGTGGACGGTT A and degenerate DBLβ degenerate reverse primer 5′- TTTRCARTACCATTCKGCCC. Products were amplified from genomic DNA extracted from a gut biopsy of patient 62 using Takara LA *Taq* DNA Polymerase (Cambrex, UK) and 35 cycles of 98°C for 15 s and 68°C for 7 min. Products were cloned using the TOPO-XL PCR Cloning Kit (Invitrogen, UK) according to manufacturer's instructions. DNA sequencing was performed at the Wellcome Trust Sanger Institute.

Identical DBL1α tags from each organ were distinguished using BLASTclust (toolkit.tuebingen.mpg.de/blastclust), defined as >95% identity at nucleotide level. Stepwise comparisons were made between tag sets from each organ and then from each patient to identify the frequency of each *var* type within organ samples and where they were shared with other organ samples and between hosts. After each unique tag was identified, sequences were aligned using clustalw (http://www.genome.jp/tools/clustalw/) and sequence data were submitted to the DDBJ/EMBL/GenBank databases under accession numbers KC678109–KC678690. Searches for identity with *P. falciparum* sequenced genomes were performed using BLAST at PlasmoDB (http://www.plasmodb.org), the Broad Institute (http://www.broadinstitute.org/annotation/genome/plasmodium_falciparum_spp/MultiHome.html) and the National Center for Biotechnology Information (http://blast.ncbi.nlm.nih.gov/Blast.cgi). All online tools used default settings. Identification of *var* group A-like sequences was performed using a perl script kindly provided by Peter Bull and described in [52]. This script identifies “group A-like” *var* tags by the presence of two conserved cysteine residues (compared to another other number between 0–6) and the presence of at least one of 573 polymorphic sequence blocks known as block-sharing group 1.

### Statistical methods

*Var* expression graphs were drawn using GraphPad Prism version 6.0 (GraphPad Software, USA) and all statistical analyses were performed using Stata version 12.0 (StataCorp, USA). Within-group comparisons of *var* group expression between individual hosts were done by analysis of variance (ANOVA) corrected by the Bonferroni-Dunn method. The non-parametric Kruskal-Wallis test was used to compare the distribution of *var* gene groups across the different clinical characteristics; age, diagnosis and HIV status. If the Kruskal Wallis test was significant, pair-wise tests were done using Mann-Whitney U test to identify groups that had a significant difference in means. Multivariate linear regression model was performed to assess association between *var* gene group expression and either age after adjusting for diagnosis or HIV status. Categorical outcomes were summarized using percentages. Fisher's exact test was used to compare proportions between two groups. All tests were declared significant if p≤0.05.

### Ethics statement

This study was approved by ethics committees at the College of Medicine, University of Malawi, Michigan State University and the Liverpool School of Tropical Medicine. Written informed consent was granted by parents or guardians of patients involved in the study.

## Supporting Information

Figure S1 Distribution of *P. falciparum* genetic variants in the organs of paediatric malaria hosts. Patients are arranged by diagnostic group (CM, cerebral malaria; PC, parasitaemic controls). A. Barcoding analysis. Each box represents a single host and each horizontal line represents a single organ as labeled on left. 24 SNPs are shown for each patient with major allele in dark grey, minor allele in light grey, heterozygous calls in orange and failed calls blank. B. *msp*2 analysis. Boxes represent hosts and organs as in A, and green shading denotes an FC27 or IC allele. Where these are vertically aligned within a patient, the genetic variants are considered identical.
(TIF)

Figure S2 Distribution of individual *var*/PfEMP1-DBL1α types in the organs of fatal paediatric malaria hosts. Each pie graph represents all DBL1α variants from a single organ of an individual host shown in the brain (A), heart (B) and gut (C). Case numbers are shown in the upper left corner of each graph and they are arranged by diagnostic group (CM, cerebral malaria; PC, parasitaemic controls). These charts are identical to those in Fig. 2 except that sections are shaded to identify DBL1α types detected in a single host (grey) or in multiple hosts (orange).
(TIF)

Figure S3 Distribution of individual *var* DBL1α types in the peripheral blood of paediatric malaria hosts. Each graph represents an individual patient. Case numbers are shown in the upper left corner of each graph. In A, sections are coloured by whether they are classified as group A-like *var* types (green) or non-group A (blue). In B, sections are shaded to identify DBL1α types detected in a single host (grey) or in multiple hosts (orange).
(TIF)

Table S1 *Var* tags that were highly expressed and/or detected in multiple patients are listed with similarity matches to the 3D7 reference genome, other *P. falciparum* genome databases and from the nucleotide sequence database at the National Centre for Biotechnology Institute.
(DOCX)

Table S2 GenBank accession numbers for all *var* sequence tags. Shading indicates in which patient and organ the tags were detected.
(XLSX)
