# Supplementary figures and images for: Differential PfEMP1 Expression Is Associated with Cerebral Malaria Pathology
Source: PLoS Pathog. 2014 Dec 4;10(12):e1004537. doi: 10.1371/journal.ppat.1004537 (PMC4256257; doi:10.1371/journal.ppat.1004537)

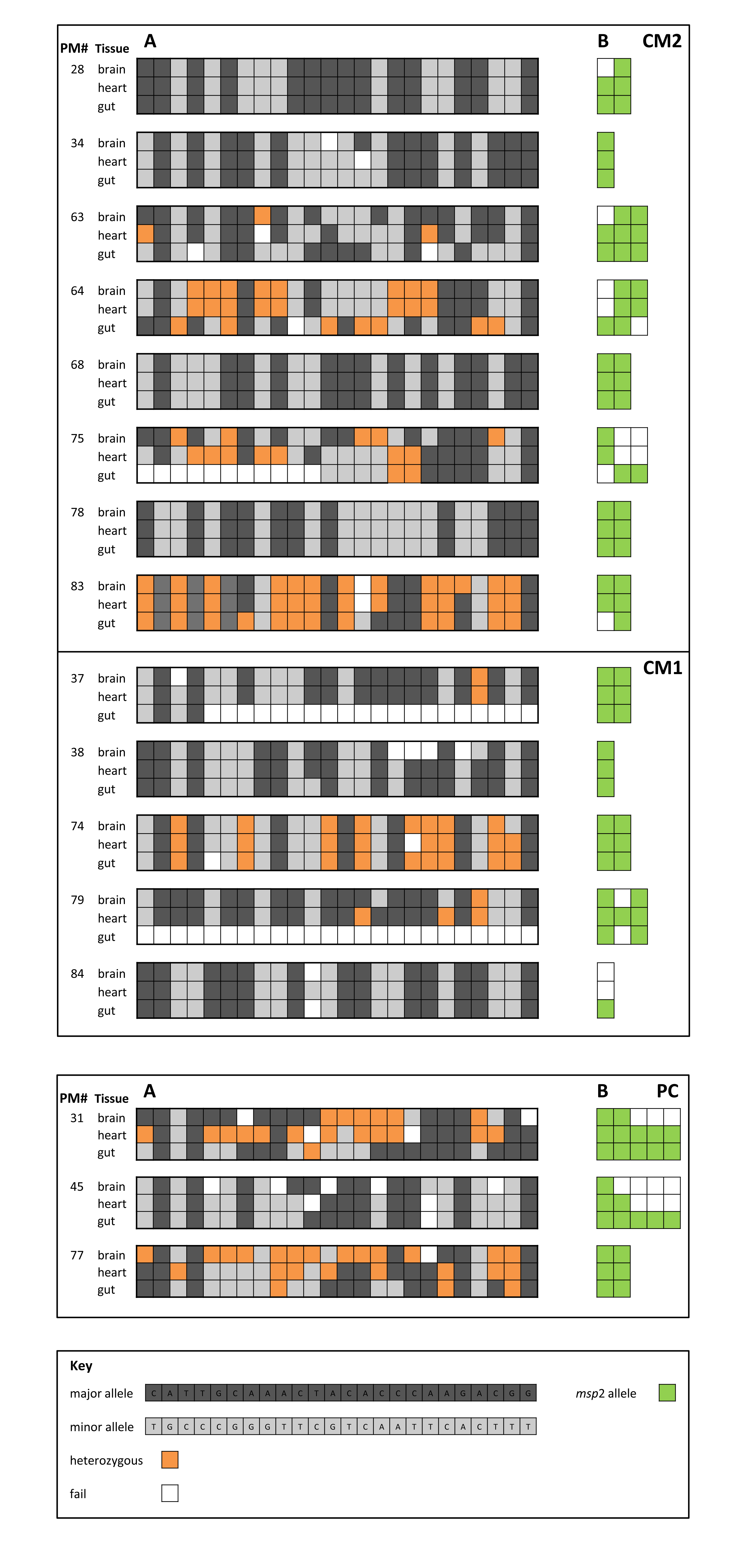

Supplement: Figure S1 — Distribution of P. falciparum genetic variants in the organs of paediatric malaria hosts. Patients are arranged by diagnostic group (CM, cerebral malaria; PC, parasitaemic controls). A. Barcoding analysis. Each box represents a single host and each horizontal line represents a single organ as labeled on left. 24 SNPs are shown for each patient with major allele in dark grey, minor allele in light grey, heterozygous calls in orange and failed calls blank. B. msp2 analysis. Boxes represent hosts and organs as in A, and green shading denotes an FC27 or IC allele. Where these are vertically aligned within a patient, the genetic variants are considered identical. (TIF) [file ppat.1004537.s001.tif]

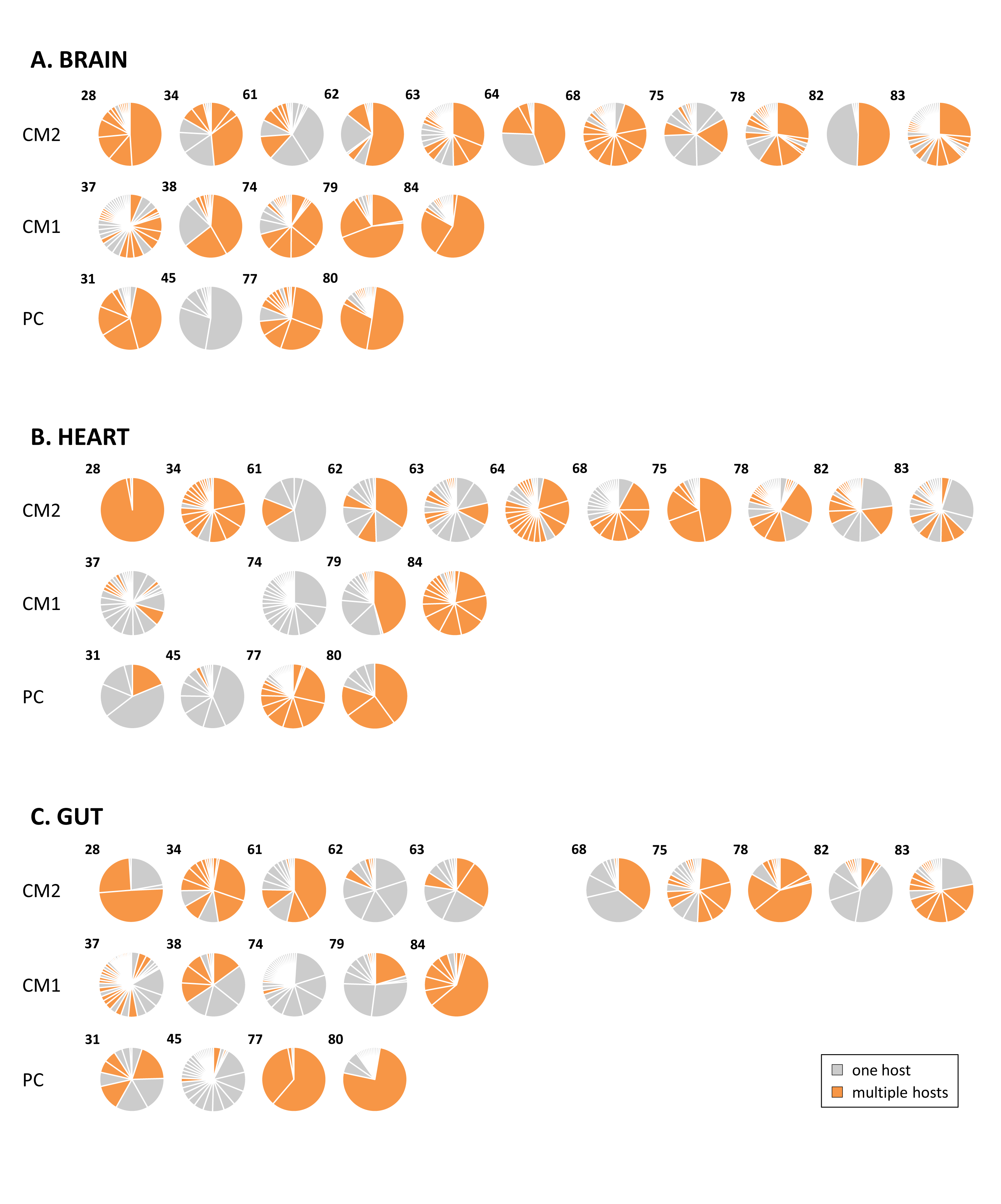

Supplement: Figure S2 — Distribution of individual var/PfEMP1-DBL1α types in the organs of fatal paediatric malaria hosts. Each pie graph represents all DBL1α variants from a single organ of an individual host shown in the brain (A), heart (B) and gut (C). Case numbers are shown in the upper left corner of each graph and they are arranged by diagnostic group (CM, cerebral malaria; PC, parasitaemic controls). These charts are identical to those in Fig. 2 except that sections are shaded to identify DBL1α types detected in a single host (grey) or in multiple hosts (orange). (TIF) [file ppat.1004537.s002.tif]

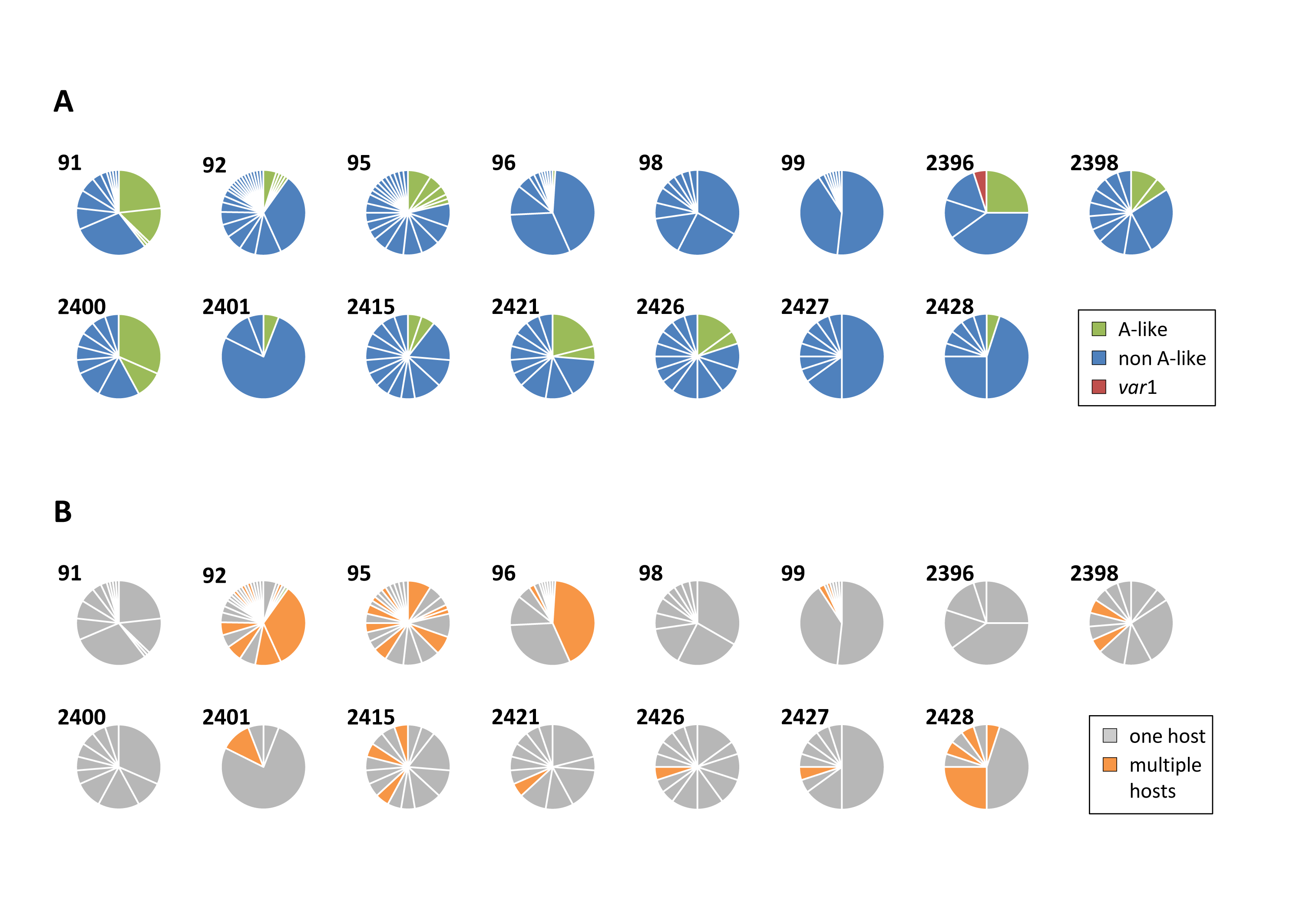

Supplement: Figure S3 — Distribution of individual var DBL1α types in the peripheral blood of paediatric malaria hosts. Each graph represents an individual patient. Case numbers are shown in the upper left corner of each graph. In A, sections are coloured by whether they are classified as group A-like var types (green) or non-group A (blue). In B, sections are shaded to identify DBL1α types detected in a single host (grey) or in multiple hosts (orange). (TIF) [file ppat.1004537.s003.tif]
