# Supplementary material for: Differential PfEMP1 Expression Is Associated with Cerebral Malaria Pathology
Source: PLoS Pathog. 2014 Dec 4;10(12):e1004537. doi: 10.1371/journal.ppat.1004537 (PMC4256257; doi:10.1371/journal.ppat.1004537)
Supplement: Table S1 — Var tags that were highly expressed and/or detected in multiple patients are listed with similarity matches to the 3D7 reference genome, other P. falciparum genome databases and from the nucleotide sequence database at the National Centre for Biotechnology Institute. (DOCX) [file ppat.1004537.s004.docx]

**Table S1: Highly-expressed DBL1α sequence tag similarity to sequence databases.**

| Tag | Accession number | *var* group | BLAST match | Identity (%) | Reference | *var* group | Genome match | Gene | Identity (%) | *var* group | 3D7 match | Identity  (%) | *var* group |
| --- | --- | --- | --- | --- | --- | --- | --- | --- | --- | --- | --- | --- | --- |
| 28B1-1 | KC678110 |  | AM115636 | 97 | a |  | RAJ116 | RAJ116var32 | 79 | B3 | PF10_0001 | 79 | B1 |
| 28B1-2.49 | KC678123 |  | HQ733027 | 71 | b |  | RAJ116 | RAJ116var06 | 81 | B1 | PFD1005c |  | B5 |
| 28E3-2 | KC678124 |  | DQ135085 | 74 | c |  | HB3 | HB3var24 | 77 | B3 | PF08_0107 |  | C1 |
| 31B2-2.02 | KC678136 |  | HQ733643 | 91 | b |  | DD2 | DD2var38 | 82 | C1 | PF08_0103 |  | B1 |
| 31B2-20 | KC678130 |  | HQ733566 | 97 | b |  | DD2 | DD2var16 | 73 |  | PFD1015c |  | C1 |
| 31E3-9 | KC678141 |  | DQ134709 | 89 | c |  | RAJ116 | RAJ116var02 | 71 | A2 | PFE1640w |  | A2 |
| 34B1-2 | KC678151 |  | DQ135087 | 73 | c |  | RAJ116 | RAJ116var08 | 82 | B1 | PFL0020w | 60 | B5 |
| 34B1-9 | KC678156 |  | HQ733014 | 68 | b |  | 3D7 | PFI1830c | 84 | B1 | PFI1830c | 60 | B1 |
| 34E3-2.34 | KC678180 |  | DQ367170 | 79 | d |  | IGH-CR14 | IGHvar38 | 77 | B1 | PF11_0007 |  | B1 |
| 34E3-27 | KC678166 |  | DQ134800 | 66 | c |  | RAJ116 | RAJ116var31 | 85 | B6 | PF13_0364 | 81 | B1 |
| 34E3-37 | KC678169 |  | HQ732590 | 73 | b |  | IGH-CR14 | PFMG_01117 | 75 |  | PFC1120c | 78 | B1 |
| 37B1-2.12 | KC678208 |  | HQ733559 | 77 | b |  | RAJ116 | RAJ116var33 | 71 | B3 | PF07_0050 | 62 | B3 |
| 38B1-2.22 | KC678248 |  | HQ733373 | 80 | b |  | IGH-CR14 | IGHvar18 | 77 | B1 | PFD1245c | 68 | B1 |
| 45B1-40 | KC678260 |  | HQ733251 | 73 | b |  | IGH-CR14 | IGHvar17 | 82 | B1 | PFD0005w | 75 | B1 |
| 61B1-27 | KC678301 |  | DQ134925 | 77 | c |  | HB3 | HB3var08 | 76 | B1 | PFD1005c | 77 | B5 |
| 61F3-2 | KC678311 |  | DQ134276 | 93 | c |  | HB3 | HB3var06 | 73 | A3 | PFL0935c | 72 | B1 |
| 62B1-1 | KC678324 | A | HQ732373 | 99 | b | A | DD2 | DD2var40 | 79 | A2 | PF13_0003 | 74 | A1 |
| 63B2-6 | KC678347 |  | HQ732559 | 81 | b |  | IGH-CR14 | PFMG_00474 | 65 |  | PFC1120c | 65 | B1 |
| 74B1-32 | KC678437 |  | HQ733207 | 72 | b |  | RAJ116 | RAJ116var09 | 75 | B1 | PFD0630c | 74 | C1 |
| 78E3-2.47 | KC678556 |  | AF221780 | 71 | e |  | RAJ116 | RAJ116var28 | 73 | C1 | PFA0005w | 73 | B1 |
| 78F3-2.25 | KC678560 |  | AF275846 | 71 | f |  | RAJ116 | RAJ116var27 | 80 | C1 | PFA0005w | 68 | B1 |
| 79B1-1 | KC678562 | A | AM116187 | 83 | a | A | IGH-CR14 | IGHvar12 | 82 | A3 | PFD0020c | 67 | A1 |

*^a^ {Bull, 2005 #556}; ^b^ {Chen, 2011 #1338}; ^c^ Barry, A.E (unpublished); ^d^ {Kyriacou, 2006 #692}; ^e^ {Taylor, 2000 #147}; ^f^ {Duffy, 2002 #182}*
